# Tailoring clinical management after embryo transfer using β-hCG levels in resource-limited settings

**DOI:** 10.1038/s41598-025-05851-y

**Published:** 2025-07-01

**Authors:** Swati Dhar, Prashanth Adiga, Anjali Mundkur, Vidyashree Ganesh Poojari, Pratap Kumar

**Affiliations:** Department of Reproductive Medicine and Surgery, Kasturba Medical College, Manipal, Manipal Academy of Higher Education, 576104 Manipal, Karnataka India

**Keywords:** Pregnancy outcome, Live birth, Embryo transfer, Biomarkers, IVF/ICSI, Quality of life, Reproductive signs and symptoms, Infertility

## Abstract

Cleavage-stage embryo transfers are often the best option for patients with limited oocytes or low fertilization rates due to medical or financial constraints. This study analyzed 424 women undergoing β-hCG testing 14 days after embryo transfer at a tertiary care center in India. Pregnancy outcomes were classified as no live births (biochemical pregnancies, ectopic pregnancies, miscarriages) or live births (single/multiple births). Higher β-hCG levels were associated with greater chances of live birth but also an increased risk of complications like preterm birth and preeclampsia, particularly in multiple pregnancies. A β-hCG threshold of 468 mIU/mL was identified as the optimal predictor of live birth, with 75% sensitivity and 72% specificity. Receiver operating characteristic (ROC) curve analysis confirmed its strong predictive value. By reducing the need for frequent monitoring, this single-test approach helps ease the emotional, financial, and logistical burdens patients face during IVF treatment. The study highlights β-hCG as a simple, cost-effective tool for providing early reassurance, guiding counseling, and personalizing follow-up, particularly in low-resource settings where access to fertility care remains challenging.

## Introduction

Serum beta-human chorionic gonadotropin (β-hCG) is a crucial marker for diagnosing early pregnancy, applicable to both natural conception and assisted reproductive technology (ART)^1^. In ART, where the cost is often prohibitive and lacks government funding, especially in middle-income countries, a reliable predictor of pregnancy success is essential. β-hCG can be detected in maternal blood as early as 8 days post-conception^2^, with its levels increasing steadily during early pregnancy. Measuring β-hCG levels 12–18 days after embryo transfer (ET) has become a common practice to assess implantation success^3^. However, the path from successful implantation to a viable pregnancy is fraught with potential complications, including miscarriage, ectopic pregnancy, or multiple pregnancies^4^. Hence, accurate early prognostication is vital for managing ART pregnancies effectively and economically. In low- and middle-income countries (LMICs), such reliable markers are particularly important due to financial, infrastructure and resource limitations that restrict repeated testing and comprehensive ART cycles^5^. The cost of an ART cycle is about 166.4% of the average annual income of a patient^6^. Consequently, many centers tend to opt for milder stimulation cycles, thus retrieving lesser oocytes, which is probably why blastocyst culture is only moderately used in such scenarios^7^. Additionally, Indian women have been noted to have a lower ovarian reserve than their European counterparts^8^. These factors, among others, lead to a high reliance on cleavage stage embryo transfers, which remain quite relevant in such settings.

Higher β-hCG levels correlate with better clinical outcomes^9^, but its predictive value remains inconsistent due to variability in the literature^1,9–12^. This variability is influenced by factors such as embryo characteristics and the timing of β-hCG measurements. Many reproductive centers standardize β-hCG testing to a fixed time post-ET to ensure more consistent comparisons within the same in vitro development period. Despite these efforts, studies have shown mixed results even with minor differences in β-hCG levels^13^. This study seeks to assess the prognostic value of β-hCG in women undergoing cleavage-stage embryo transfer, aiming to clarify its effectiveness as a predictor of reproductive outcomes in the context of ART.

## Results

The sociodemographic and cycle characteristics of the study groups are shown in Table 1 and were comparable in both the groups. Only β-hCG levels were significantly higher in those who had a live birth when compared to those who had no live birth (1112 *±* 867.6 mIU/ml vs. 448.4 *±* 630.2 mIU/ml, *p* < 0.001). The mean age of the patients in both the groups was on the higher side, which also highlights delayed treatment seeking behavior, possibly due to social and financial factors.

**Table 1:** Demographics and cycle characteristics of the live birth group (*n* = 274) and no live birth group (*n* = 150).

Variables	Groups		
	Live birth (*n* = 274)	No live birth (*n* = 150)	*p*-value
	Mean *±* SD	Mean *±* SD	
Age (years)	34.44 *±* 5.06	34.89 *±* 5.21	0.385
βhCG (mIU/mL)	1312.31 *±* 2289.33	638.71 *±* 2346.4	0.0042
	n (%)	n (%)	
Cause of infertility Female factor Male factor Combined Unexplained	62 (22.7%) 115 (42.22%) 67 (23.89%) 30 (11.02%)	40 (26.31%) 62 (40.78%) 34 (23.68%) 14 (9.21%)	0.833
Embryo transferred Autologous oocyte Donor oocyte	171 (60.8%) 103 (70.6%)	110 (39.14%) 40 (29.4%)	0.054
Type of transfer Fresh Frozen	67 (73.6%) 207 (61.5%)	26 (26.4%) 124 (38.4%)	0.086
Endometrial thickness (mm) Q1 (< 8.38) Q2 (8.4–9.2) Q3 (9.2–10.4) Q4 (> 10.4)	9.59 *±* 1.35 62 (57.54%) 74 (69.81%) 65 (60.37%) 73 (68.86%)	9.38 *±* 1.56 44 (42.46%) 32 (30.19%) 41 (39.63%) 33 (31.14%)	0.166 0.159

Continuous variables presented as Mean *±* SD and analyzed Mann Whitney U test. Categorical variables presented as percentage and analyzed using Chi square test. Significant differences are indicated (*p* < 0.05).

The β-hCG values were compared between fresh and frozen embryo transfers, as well as between self and donor oocyte groups, and no statistically significant differences were found (Table 2). This indicates that β-hCG levels are comparable across these groups, allowing them to be analyzed collectively as a single statistical variable in embryo transfer outcomes.

**Table 2:** Beta-hCG values in fresh/frozen and self/donor embryo transfers in our study:.

	Beta hCG (Mean *±* SD)	*p*-value
Self	810.8 *±* 802.8	0.068
Donor	937.0 *±* 785.1	
Fresh	943.0 *±* 852.3	0.147
Frozen	799.2 *±* 764.3	

Continuous variables presented as Mean *±* SD and analyzed using Mann Whitney U test. Significant differences are indicated (*p* < 0.05).

**Fig. 1:**
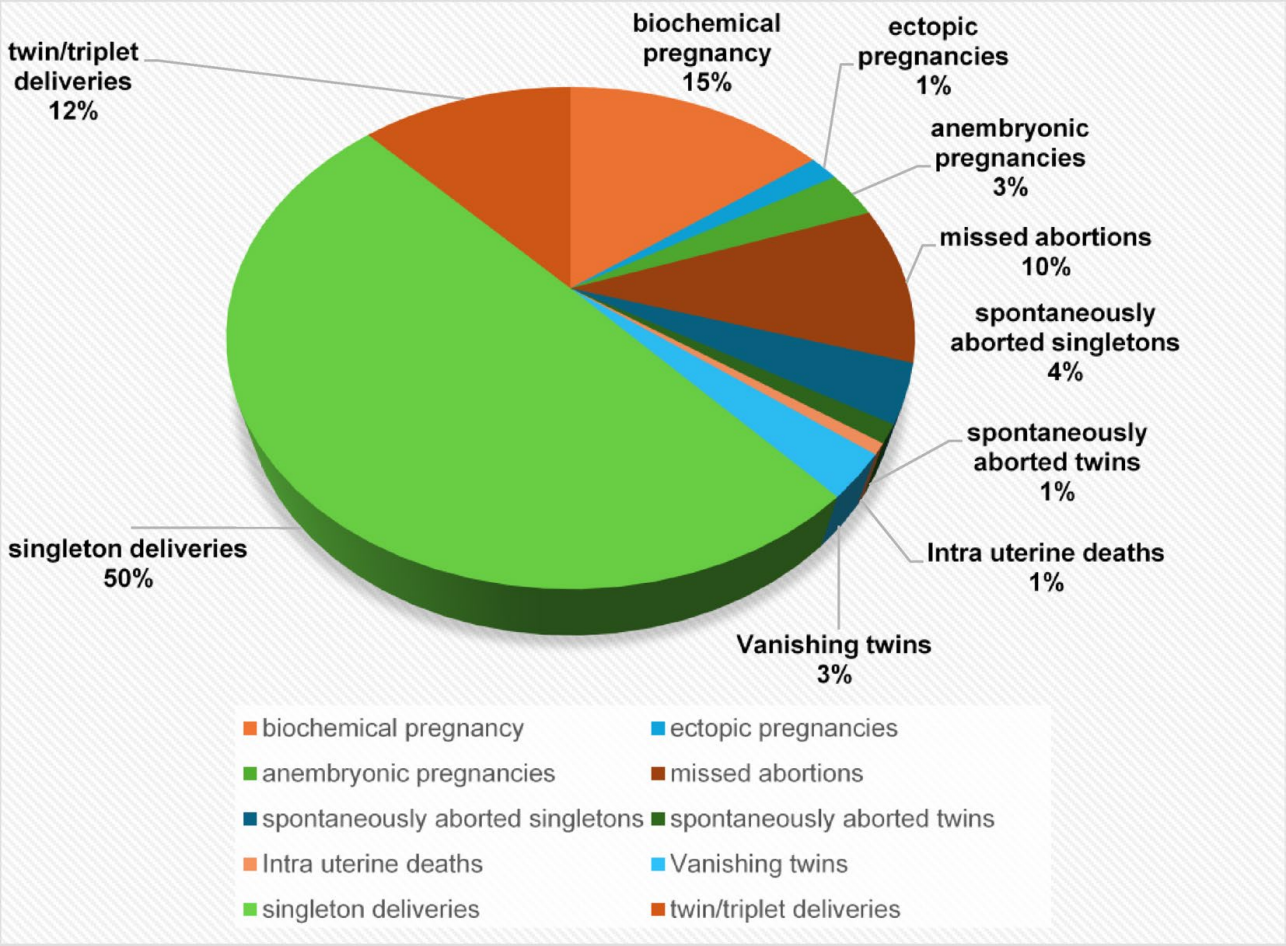
Pregnancy outcomes in study group (*N* = 424).

Figure 1 depicts in detail the pregnancy outcomes in the study cohort. Those with no live birth group comprised of 63 biochemical pregnancies, 7 ectopic pregnancies, 13 anembryonic pregnancies, 43 missed abortions, 16 spontaneously aborted singletons, 5 spontaneously aborted twins or triplets and 3 had intrauterine fetal deaths. Among the women in the live birth group 12 had vanishing twins, 211 had singleton deliveries, and 51 had twin/triplet deliveries.

**Fig. 2:**
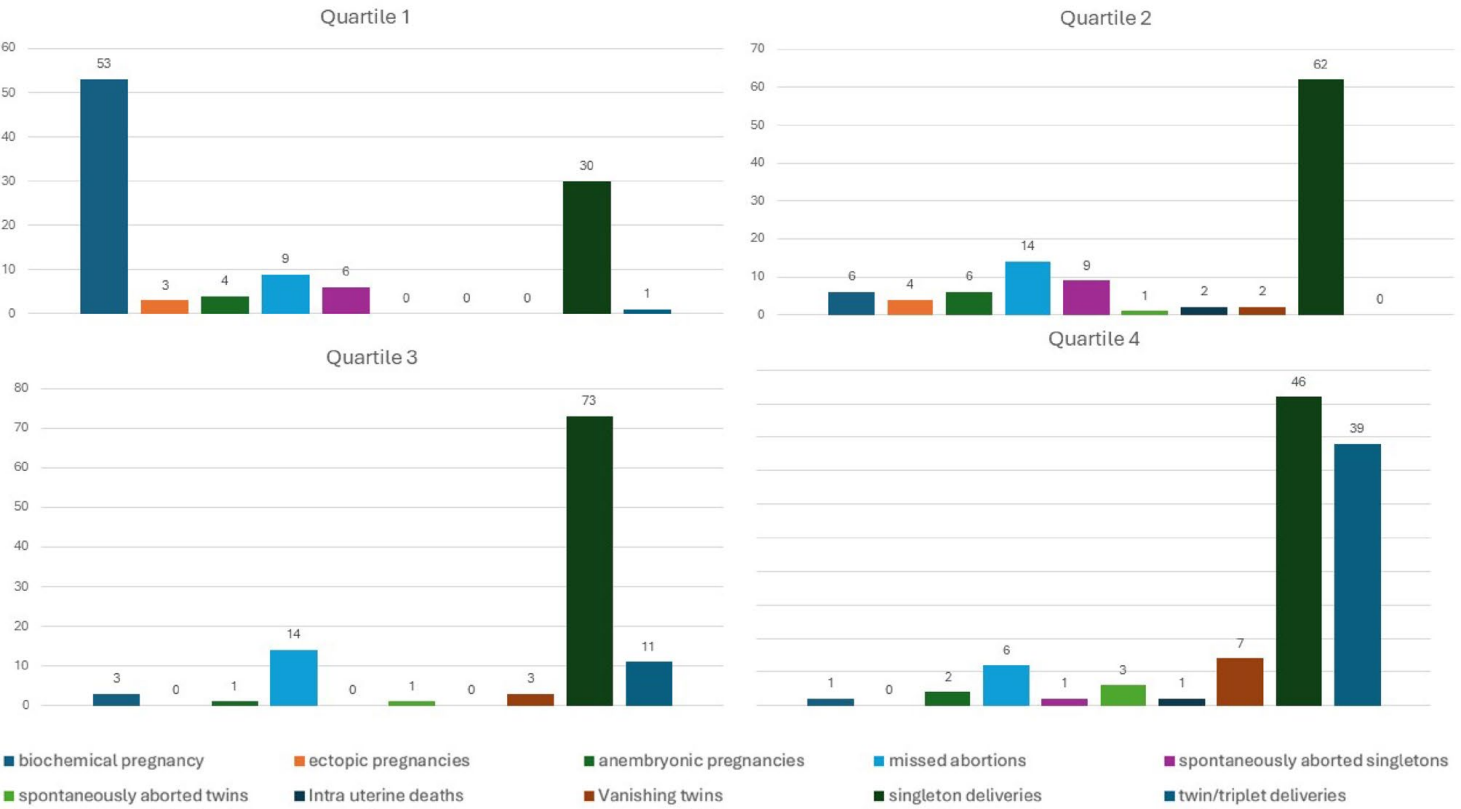
Figure showing the Comparative Analysis in quartile groups of βhCG in different pregnancy outcomes in our study (*n* = 106 in each quartile).

β-hCG values ranged from 5.2 mIU/ml to 3713 mIU/ml. They were further divided into quartiles of Q1, Q2, Q3 and Q4, with values of less than 226, 226 to 595, 595.1 to 1317, and more than 1317mIU respectively. This was done to analyse trends, and especially to detect non-linearity of β-hCG values with respect to various pregnancy outcomes across these quartiles. We found that with increasing quartiles, the number of biochemical pregnancies significantly decreased, whereas the number of multiple pregnancies and live births significantly increased. The number of nonviable pregnancies had a moderately negative trend from lower to higher quartiles, as shown in Fig. 2.

**Fig. 3:**
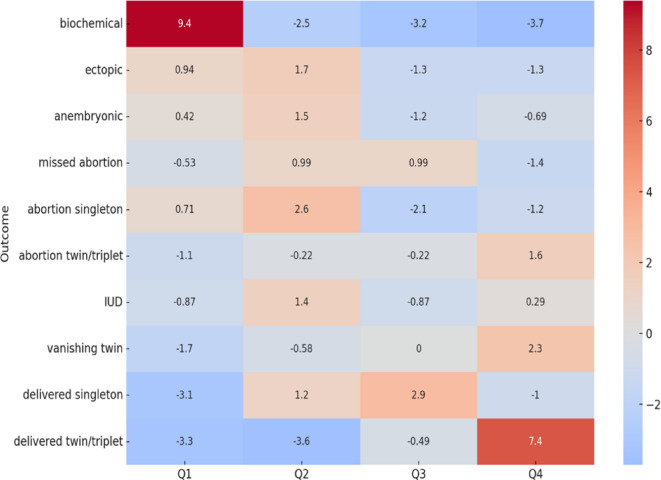
Residual Heat map of a post-hoc analysis test done after Chi-square test in different quartiles of β-hCG values.

Chi-square test was performed on the different pregnancy outcomes and was found to be significant (chi-square value: 261.74, (df): 27 p value: 2.72 × 10–40). This was followed by a post hoc residual analysis. These findings are summarized in the residual heatmap depicted below (Fig. 3).

The Q1 and Q4 quartiles were highly predictive of biochemical pregnancies and multiple gestation deliveries, respectively. However, the Q4 quartile also had several complications, mostly spontaneous abortions associated with multiple pregnancies. Ectopic and anembryonic pregnancies were mostly confined to the lower quartiles. However, missed abortions were equally common in the middle 2 quartiles, which is important to note, as chances of a singleton delivery are also highest in the same quartiles, albeit with a higher probability. Interestingly, of the 3 IUDs reported, 2 belonged to the lower quartile (Q2) and had fetal growth restriction (FGR) as the underlying cause. The remaining one belonged to the highest quartile (Q4) and was a case of severe preeclampsia. The women in the live birth group were further analysed for feto-maternal complications. The incidence of threatened abortion showed a decreasing trend across the quartiles, as expected. Notably, complications were more prevalent in higher quartiles, particularly gestational diabetes (GDM), preeclampsia (PE), prelabor rupture of membranes (PROM) and preterm birth, mainly owing to the higher incidence of twins. The post-hoc power analysis demonstrated strong statistical power for all outcomes except FGR, indicating reliable detection of significant differences where they exist, as shown in Table 3.

**Table 3:** Feto-maternal complications in those who gave live birth (*n* = 274).

β-hCG quartiles	Complications							
	Threatened abortion (*n* = 53)	GDM (*n* = 44)	PE (*n* = 41)	APH (*n* = 16)	FGR (*n* = 23)	PROM (*n* = 26)	Term (*n* = 144)	Preterm (*n* = 130)
Q1 (< 226) (*n* = 31)	11 (35.5)	3 (9.6)	3 (9.6)	1 (3.2)	4 (12.9)	3 (9.6)	13 (41.9)	18 (58.1)
Q2 (226–595) (*n* = 64)	13 (20.6)	11 (17.5)	4 (6.3)	2 (3.2)	6 (9.5)	5 (7.9)	46 (71.4)	18 (28.6)
Q3 (595.1–1317) (*n* = 87)	16 (18.4)	17 (19.5)	16 (18.4)	8 (9.2)	7 (8)	4 (4.6)	52 (59.8)	35 (40.2)
Q4 (> 1317) (*n* = 92)	13 (14.3)	13 (14.3)	18 (19.8)	5 (5.5)	6 (6.6)	14 (15.4)	33 (35.2)	59 (64.8)
Chi^2^ Statistic	21.13	9.45	18.02	7.5	0.8	11.85	24.82	34.74
P-Value	**< 0.001**	**< 0.024**	**< 0.001**	0.058	0.84	**0.008**	**< 0.001**	**< 0.001**

Values presented as frequencies (percentages) analyzed with chi-square test, ^a^Fischer’s exact test, p-value significant at < 0.05. Abbreviations: (GDM- Gestational Diabetes Mellitus, PE- Preeclampsia, APH- Antepartum haemorrhage, FGR- Fetal growth restriction, PROM- Prelabour rupture of membranes).

### Receiver operating curve (ROC) analysis

**Fig. 4:**
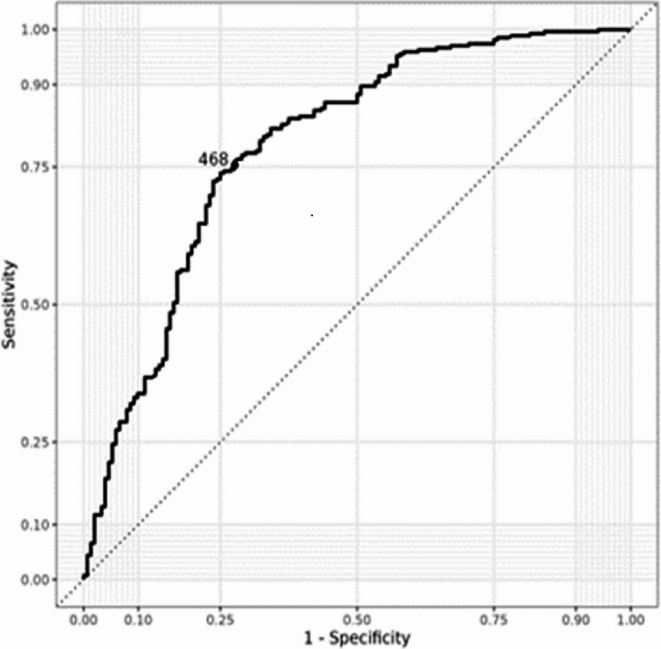
Receiver operating characteristic (ROC) curve for prediction of live birth based on serum values of β-hCG.

We conducted a logistic regression to calculate the ROC curve analysis which was used to explore the predictive value of serum β-hCG levels for the possibility of live birth (Fig. 4). The area under the curve (AUC) for the β-hCG level was 0.788, which was statistically significant. The β-hCG threshold value for predicting pregnancy outcome via Youden’s index algorithm in the ROC curve was 468 mIU/mL, with a specificity of 72% and a sensitivity of 75%. The Positive Predicitve value of the cut-off is 83%, while the Negative Predicitve Value is 61.2%.

## Discussion

In our study, we sought to determine the relevance of β-hCG for predicting and prognosticating pregnancy outcomes in patients undergoing IVF. A single β-hCG measurement on day 14 post-ET strongly predicts clinical pregnancy outcomes, particularly live births, with a cutoff of 468 mIU/ml. Given the high positive predictive value (83%) of the β-hCG cut-off, clinicians may consider limiting further β-hCG measurements or deferring early transvaginal ultrasonography in asymptomatic IVF patients, reducing both patient burden and healthcare utilization. This approach eliminates the need for repeated β-hCG measurements, offering significant financial and psychological benefits, particularly in middle-income countries where healthcare costs and accessibility are key concerns. Higher β-hCG levels were associated with multiple gestations, consistent with previous findings^12,14^. The fresh or frozen embryo transferred in the studied population had comparable beta hCG values corroborating the findings of other studies done by Reljič et al.^11^ and Lawler et al.^15^. Some studies have shown an increase in beta hCG in fresh embryo transfer to be more than frozen transfers like Shibli et al.^16^, but they have taken into consideration both cleavage stage and blastocyst transfers, that may affect the results.

Unlike other studies, such as those by Ozer et al.^17^ and Trautner et al.^18^, which focused on blastocyst transfers, our study uniquely targeted cleavage-stage transfers. While the trend in ART is shifting toward blastocyst transfers, cleavage-stage transfers are still common, especially in cases with fewer oocytes, making our results highly relevant^19^. Also, in LMICs the use of blastocysts for transfers is still moderate at best, as highlighted in a recent review^7^. In contrast to Wang et al.^9^, who measured β-hCG on day 7 to predict early pregnancy outcomes, we measured β-hCG on day 14, offering a more practical and standardized approach. Moreover, the late pregnancy complications were also reported and analysed, reflecting the role of hCG values far beyond the first trimester. Unlike previous studies such as Sung et al.^12^and Shibli et al.^16^ which focused only on singleton deliveries, our study included multifetal pregnancies to provide a broader and more clinically relevant perspective on IVF outcomes. Given that double embryo transfer remains a common practice, excluding multiple pregnancies would not fully capture the real-world burden and risks associated with ART. While we acknowledge that multifetal pregnancies may introduce variability, our analysis accounts for potential confounders, ensuring that the findings remain robust. Moreover, despite double embryo transfers, the multiple pregnancy rates remained relatively low in our population. This approach of inclusion enhances the generalizability of our study by reflecting the full spectrum of IVF outcomes encountered in clinical settings.

Previous studies have shown an association between maternal age and pregnancy loss, particularly in women over 35 years of age, due to diminished follicle reserves and increased chromosomal abnormalities^20–22^. However, our study did not find a statistically significant difference in the ages of women who delivered versus those who experienced pregnancy loss. This finding may be explained by the fact that many older women in our study received donor oocytes, which tend to be of higher quality, thus compensating for the maternal age^23^. Our results align with those of Singh et al.^24^, who correlated maternal age and β-hCG levels on day 14 post-ET with ongoing pregnancy outcomes. While our study did not focus exclusively on maternal age, it has been noted that β-hCG levels tend to be higher in younger women, possibly due to better oocyte quality and greater endometrial receptivity^25–27^.

There was no significant difference observed in pregnancy outcomes or β-hCG levels between cycles using donor oocytes and those using self-oocytes, aligning with findings from Hughes et al.^28^. This insight is valuable as donor oocyte cycles are often underrepresented in studies on assisted reproductive technology (ART). Similar to the inclusion of multifetal gestation, it provides a more realistic representation of real-world scenarios, while the statistical analysis attempts to decrease the associated confounding, thus providing practical results. The comparable results in donor versus self-cycles can likely be attributed to careful selection of candidates for either cycle. Factors such as the patient’s age, ovarian reserve, and anticipated response to ovarian stimulation are crucial in making this decision, ensuring that both approaches have a similar likelihood of success.

One of the strengths of our study is its large sample size, which allowed us to derive a reliable β-hCG cutoff value for live birth prediction that can be applied to both fresh and frozen embryo transfers. Unlike Grin et al.^29^, who suggested different cutoffs for fresh and frozen cycles with values of 211 IU/L and 440 IU/L respectively, we propose a single cutoff of 468 mIU/ml, simplifying clinical practice. Our findings are also consistent with those of Zhang et al.^30^, who reported significantly higher β-hCG levels in live births compared to miscarriages (596.8 mIU/mL vs. 357.15 mIU/mL; *P* < 0.001) and Hu et al.^31^, who found higher β-hCG levels in live births without determining a specific cutoff. An unexpected finding was the relatively high number of abortions observed in the higher β-hCG quartiles (Q3 and Q4), despite the association of higher β-hCG with better pregnancy outcomes. This could be attributed to probable chromosomal abnormalities or the higher rate of multiple gestations in this group, as suggested by a previous study^32^. While β-hCG is a strong predictor of clinical pregnancy, it cannot fully account for the complexities of pregnancy outcomes, particularly in cases of aneuploidy or multifetal gestations, which may contribute to higher morbidity, as observed with conditions like preeclampsia. The frequencies of feto-maternal outcomes observed in our study were in accordance with a much larger study^33^, suggesting the need for vigilance at both extremes and thus highlights the necessity of careful interpretation even at acceptably good levels of hCG.

Our study holds significant implications especially for middle-income countries, where IVF is becoming more accessible but remains costly, largely privatized, and often uninsured^7^. The identification of a reliable β-hCG threshold has significant clinical implications. In patients who are clinically asymptomatic and meet the cut-off criteria, repeated β-hCG measurements and early ultrasound scans may be unnecessary. This reduces the financial burden on patients, alleviates anxiety associated with frequent monitoring, and limits unnecessary clinic visits without compromising pregnancy outcomes. Such an approach may be particularly valuable in resource-constrained settings where access to repeated investigations is limited. Moreover, adopting a more streamlined follow-up protocol may improve efficiency in high-volume fertility centers.

We confirmed that β-hCG is a valuable predictor of live births and established a single cutoff applicable to both fresh and frozen embryo transfers. However, certain unanswered questions remain particularly regarding the influence of chromosomal abnormalities and multifetal pregnancies on outcomes, even at higher β-hCG levels. Importantly, while an elevated β-hCG level indicates a higher likelihood of live birth, it does not always correlate with optimal feto-maternal outcomes, reinforcing the need for ongoing prenatal care. One limitation of our study is the lack of ploidy status data, which could influence pregnancy outcomes, though this remains an inherent challenge in cleavage stage embryo transfers. A key strength is the large cohort size, encompassing both fresh and frozen cycles, as well as self and donor oocytes, ensuring a diverse and representative population. A key limitation of this study is the exclusion of patients with poor-quality embryos, which may limit its generalizability to broader IVF populations. However, this exclusion was necessary to minimize confounding, as poor embryo quality is an independent predictor of adverse outcomes, particularly in cleavage-stage transfers. By focusing on good-quality embryos, we aimed to enhance the interpretive precision of β-hCG as a predictive tool. The use of electronic medical records further enhanced data accuracy despite the study’s retrospective nature.

Our findings reinforce the clinical utility of early β-hCG estimation in prognosticating pregnancy outcomes and guiding patient counseling. However, interpretation must be pragmatic, while lower values allow for early identification of adverse outcomes, higher values do not necessarily ensure favorable maternal or fetal results. Thus, it must also be noted that higher values of initial β-hCG (in this study, Q4, i.e., 1317 mIU/mL) may not always indicate optimal feto-maternal outcomes. Thus, this non-linearity of βhCG with a single-point outcome of any cutoff value should be interpreted with caution. Therefore, β-hCG assessment should be complemented by sonography and comprehensive prenatal care to optimize pregnancy management. A prospective study will be undertaken to confirm whether the β-hCG cutoff level predicts outcomes. We propose that our findings serve as a foundation for future large-scale, multicenter studies with standardized methodologies, particularly in settings with limited access to repeated advanced testing, to refine β-hCG cutoffs and enhance their applicability across diverse clinical contexts.

## Methods

This single-centric retrospective cohort study was conducted in the Department of Reproductive Medicine and Surgery, Kasturba Medical College Manipal, a tertiary care hospital in Karnataka, India during March 2018 to August 2022. The study was conducted in accordance to the institutional guidelines. The Kasturba Medical College and Kasturba Hospital Institutional Ethics Committee waived consent, as the study was retrospective in nature (IEC1:205/2023). However, permission was obtained from the hospital authorities before accessing medical records. Only those with a positive serum β-hCG (> 5 IU/L) at 14 days^34^ after good quality (grade1/2 as per Istanbul Consensus)^35^ cleavage stage embryo transfers were included.

Both donor and autologous oocytes were included in the study. Exclusions comprised of those who had *≥* 3 embryos transferred, untimely β-hCG, mullerian anomalies, endometrial thickness < 7 mm, presence of fibroid distorting endometrial cavity, who did not follow-up antenatally at our center, those with fetal anomalies, preimplantation genetically tested embryos, recurrent pregnancy loss and those with incomplete data.

### Data collection and follow-up

Nine seventy-five cleavage stage ETs were performed, of which 793 had beta hcg estimation 14 days post transfer at our standardized laboratory.

**Fig. 5:**
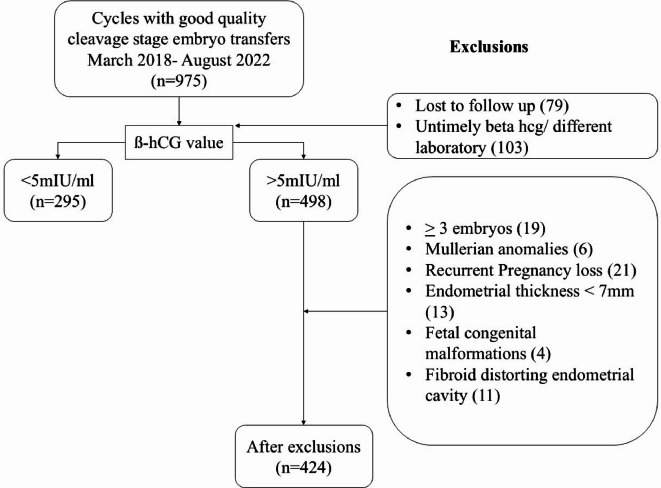
Flow diagram of study cohort (*N* = 424).

Out of these, 498 β-hCG positive patients were screened for exclusion criteria. A total of 424 patients were finally included in the analysis, as shown in Fig. 5. It was planned to exclude PGT embryos from the analysis. However, no genetically tested embryos were transferred during the period. Sociodemographic data and relevant clinical data were collected using electronic medical records.

The details regarding treatment protocol used in the study population are as follows:

### IVF stimulation protocol

Controlled ovarian stimulation was performed via either the antagonist (AMH > 1.5 ng/ml) or the microdose flare protocol (AMH < 1.5 ng/ml). The dosage and type of gonadotrophin were determined based on patient characteristics and physician preferences. Biochemical and ultrasonographic cycle monitoring were performed. Oocyte retrieval was performed under short general anaesthesia, 35 h after the oocyte maturation trigger (recombinant hCG or gonadotrophin releasing hormone agonist (GnRHa)). IVF/ICSI was performed according to laboratory protocols.

On Day 3, all the embryos were evaluated according to the Istanbul consensus grading system^35^. The top-quality embryos were either transferred fresh or vitrified for future use, depending on the cycle and patient characteristics.

### Embryo transfer preparation

Artificial cycle frozen embryo transfers (AC-FETs) for autologous embryos were performed after midluteal GnRHa-induced pituitary downregulation in the previous cycle. On day 2 of the menstrual cycle, transvaginal sonography (TVS) was performed, after which 2 mg of oral estradiol valerate was started thrice daily. TVS was repeated after one week, and the dose was increased if needed. Progesterone intravaginal gel was started once a thickness of at least 7 mm was achieved after a minimum of 14 days of estradiol valerate. Embryo transfer was performed on day 4 after the start of progesterone gel.

Fresh embryo transfers were performed for suitable candidates on day 4 of progesterone treatment, which started after oocyte retrieval. For donor cycles, recipients receive estrogen followed by progesterone to synchronize their cycles and endometrium with the donor, followed by a fresh embryo transfer. Subsequent donor frozen transfers followed the same protocol as described for autologous frozen transfer preparation.

### Embryo transfer and luteal support

As per our unit protocol, two or more day 3 embryos were transferred under sonographic guidance. Cleavage stage embryos were preferred in these patients due to lesser retrieved oocytes and also as per the unit’s clinician-embryologist consensus on the modest implantation potential of two cleavage stage embryos compared to single blastocyst transfer, while decreasing chances of cycle cancellation compared to blastocyst cycles^36^. In India, the law permits use of only seven retrieved donor oocytes, hence cleavage stage transfers are often preferred over extended blastocyst culture^37^. Intensive luteal phase support was provided following transfer. This included progesterone via the intravaginal and intramuscular routes, along with oral dydrogesterone and estradiol valerate. In addition, a single dose of GnRHa (0.1 mg triptorelin) was given at 3 days post transfer. Moreover, all women were screened for Antiphospholipid Antibody Syndrome and received thromboprophylaxis with Inj Enoxaparin 0.4 mg- 0.6 mg daily post transfer if positive. None of them received hCG injections as LPS.

### β-hCG evaluation and follow-up

Serum β-hCG was assessed 14 days post transfer via an electrochemiluminescence immunoassay. All β-hCG-positive pregnancies (> 5 mIU/mL) were followed up until the pregnancy outcome in the same unit.

Pregnancy outcomes were then categorised as “live birth” or “no live birth”. “No live births” included biochemical pregnancy, ectopic pregnancy, anembryonic pregnancy, missed abortion, intra uterine deaths and spontaneous abortion, whereas pregnancies that delivered were considered as “live birth”.

### Outcome variables

The primary aim was to analyse the trends of various pregnancy outcomes across different β-hCG values. The secondary outcome of our study focused on analysing the trend of fetal–maternal complications such as gestational diabetes (GDM), preeclampsia (PE), prelabor rupture of membranes (PROM) and preterm birth in those who had a continuation of pregnancy. A cut-off value of serum β-hCG was also determined to predict the possibility of a live birth.

### Ethics approval

The study was approved by the Kasturba Medical College and Kasturba Hospital Institutional Ethics Committee with number IEC1:205/2023. The Committee exempted the authors from obtaining individual informed consent due to the retrospective nature of the study.

### Statistical methods

GraphPad Prism version 10.2.3 for Windows (GraphPad Software, Boston, Massachusetts, USA; www.graphpad.com) was used to analyse the data. Continuous quantitative data were first analysed via the Shapiro‒Wilk normality test and are presented as the means ± standard deviations (SDs), medians, and quartiles. Student’s t test and an unpaired t test with Welch’s correction were used for normally distributed and non-normally distributed data, respectively. Categorical data are presented as percentages and were compared via the chi-square test or Fischer’s exact test. A post-hoc Chi-square residual analysis and power analysis were also carried out. Furthermore, the serum β-hCG levels were analysed via receiver operating characteristic (ROC) curves. All tests were two-sided, and a p value of < 0.05 (95% CI) was considered to indicate statistical significance.

## Data Availability

The datasets used and/or analyzed during the current study are available from the corresponding author on reasonable request.
